# Comparison of clinical efficacy of suprapatellar and infrapatellar intramedullary nailing in treating tibial shaft fractures

**DOI:** 10.12669/pjms.37.7.4766

**Published:** 2021

**Authors:** Zhonglian Zhu, Zhaodong Wang, Pinghui Zhou, Xuyi Wang, Jianzhong Guan

**Affiliations:** 1 Zhonglian Zhu, Anhui Key Laboratory of Tissue Transformation, Bengbu Medical College, Department of Orthopedics, The First Affiliated Hospital of Bengbu Medical College, 287 Changhuai Rd, Bengbu 233000, Anhui Province, P.R. China; 2 Zhaodong Wang, Department of Orthopedics, The First Affiliated Hospital of Bengbu Medical College, 287 Changhuai Rd, Bengbu 233000, Anhui Province, P.R. China; 3 Pinghui Zhou, Department of Orthopedics, The First Affiliated Hospital of Bengbu Medical College, 287 Changhuai Rd, Bengbu 233000, Anhui Province, P.R. China; 4 Xuyi Wang, Department of Orthopedics, The First Affiliated Hospital of Bengbu Medical College, 287 Changhuai Rd, Bengbu 233000, Anhui Province, P.R. China; 5 Jianzhong Guan, Anhui Key Laboratory of Tissue Transformation, Bengbu Medical College, Department of Orthopedics, The First Affiliated Hospital of Bengbu Medical College, 287 Changhuai Rd, Bengbu 233000, Anhui Province, P.R. China

**Keywords:** Intramedullary nail, Tibial shaft fracture, Suprapatellar approach, Infrapatellar approach

## Abstract

**Objectives:**

To compare clinical efficacies of suprapatellar and infrapatellar intramedullary nailing approaches in treating tibial shaft fractures.

**Methods:**

Patients (n=110) admitted with tibial shaft fractures in our hospital from January 2017 to June 2020, who underwent procedures with internal fixation intramedullary nails, were retrospectively divided into suprapatellar and infrapatellar approach groups (n = 55 each) based on the surgical method used for fracture repair. The clinical and functional outcomes of the knee were assessed six months after the surgery

**Results:**

Six months after the operation, the pooled value for excellent and good efficacy rates in the suprapatellar approach group, as indicated by Hospital for Special Surgery (HSS) Knee scoring system, was 90.91%, which was significantly higher than that in the infrapatellar approach group (76.36%). The degree of pain (visual analogue scale (VAS) score) of the patients in the suprapatellar approach group was over 2-fold lower than in the infrapatellar approach group (P < 0.001).The Lysholm knee score, range of motion (ROM), SF-36p, and SF-36M scores in the suprapatellar approach group were significantly higher than those in the infrapatellar approach group (P < 0.001).

**Conclusion:**

Suprapatellar approach had significantly higher clinical efficiency than infrapatellar approach, and can significantly reduce the degree of pain, promote the recovery of patients with knee joint involvement, improve the physical and psychological well-being, reduce the number of cases of postoperative delayed healing

## INTRODUCTION

Tibial fracture is a common long bone fracture, mostly caused by high-energy impacts. After a tibial fracture, the weight bearing capacity of the affected limb is lost and, if not treated in time, it seriously affects the quality of life of patients with pain and limping among other symptoms.^1^,^2^ The most common treatment for tibial fracture is intramedullary nail fixation to promote the recovery of the affected limb. Intramedullary nailing techniques include the suprapatellar and infrapatellar approaches. Intramedullary nailing through the infrapatellar route is the traditional technique, and the resulting internal fixation is relatively strong, allowing for the early exercise of patients; however, the operation is difficult, the patient’s position needs to be changed repeatedly, and the tibial shaft fracture reduction effect is imperfect.^3^,^4^ The approach is a new operative method. This intramedullary nailing technique has gained acceptance for tibial shaft fractures, but the sharp instruments used during the operation may damage the articular surface and cartilage of the patella, and whether this or the infrapatellar technique has better specific curative effects is in dispute.^5^,^6^ This study was designed to compare the efficacy and prognosis of supra- and infrapatellar intramedullary nailing techniques for the treatment of tibial shaft fractures.

## METHODS

This retrospective study included data from the records of 110 patients admitted to The First Affiliated Hospital of Bengbu Medical College hospital with tibial shaft fracture from January 2017 to June 2020. Patients were divided into suprapatellar and infrapatellar approach groups based on the surgical procedure performed (55 cases in each group). The demographic data of the patients in the two groups is shown in Table-I. The two groups of patients had similar basic background information (P > 0.05). Inclusion criteria were as follows: patients were diagnosed by X-ray as having unilateral tibial shaft fractures that could be treated with closed reduction and intramedullary nailing; fractures had occurred within 24 hours of the hospital admission; participants could tolerate the operation. Exclusion criteria were as follows: patients with incomplete clinical data or lost to follow-up; lactating or pregnant women; patients with pathological or old fractures; those with severe cardiovascular, cerebrovascular diseases, mental illness, or inability to communicate. The study was approved by the Ethics Committee of The First Affiliated Hospital of Bengbu Medical College.

**Table I:** Comparison of basic data of patients in the two groups.

*Information*		*Suprapatellar approach group (n = 55)*	*Subpatellar approach group (n = 55)*	*T/χ^2^ value*	**P* value*
Gender (M/F)		28/27	30/25	0.146	0.703
Average age (years)		32.1±4.2	33.0±3.8	1.178	0.241
Time from fracture to admission (hours)		16.2±3.2	15.9±2.9	0.515	0.607
Cause of injury	Road Traffic Injury	26	25	0.482	0.923
	Fall	12	14		
	Blow	8	9		
	Other injuries	9	7		

In-between groups analysis was done using the T test and a single factor analysis of variance was performed to compare multiple groups. Measurement data are pressed as means and standard deviations (x ± s). Enumeration data are expressed as X^2^ test results.

For the intramedullary nailing procedure through the suprapatellar approach, after general anesthesia with the patients laying down in a supine position, a knee was flexed to about 20°, and a 5 cm incision was made 2 cm proximal to the superior pole of the patella to obtain the desired nailing entry point with the guidance of C-arm image intensifier. The surgeon then blunt-dissected the quadriceps tendon and suprapatellar bursa lengthwise, placed the guide wire and protective sleeve along the axis of tibial medullary cavity, and set the medial side of the lateral tibial spine as the entry point with the guidance of C-arm image intensifier. Intramedullary nails of appropriate length (Anhui Guoke Hengtai Medical Technology Co., Ltd.) were placed in the tibia, and two locking nails were placed in the proximal hole with the specialized insertion cannula as per convention. Two locking screws were put in the distal holes and then the corresponding tail caps were placed, and the alignment was assessed. Before closure the articular cavity was repeatedly washed.

For the intramedullary nailing procedure through the infrapatellar approach, after the general anesthesia induction, the patients in a supine position had the affected limb raised 15 cm, and the fracture location was determined under fluoroscopy. The surgeon then performed a 6-cm incision longitudinally from the apex of patella to the tibial tubercle, and separated the patellar ligament using a sharp dissection technique. At the upper slope of the tibial tuberosity, the surgeon inserted a long guide wire along the axis of the tibial medullary cavity. Later the fracture was reduced closed under C-arm control. The reduction was maintained by a guide pin inserted into the distal metaphysis of the tibia. Intramedullary nails were inserted into the tibia with the assistance of a C-arm machine and locking screws were inserted distally and proximally. After fixation, the surgeon cleaned the wound, and sutured the incision.

### Postoperative treatment was as follows

One or two days after the operation, the patients were given antibiotics; 3 to 5 days after the operation, the patients underwent physiotherapy without weight bearing. Weight bearing gradually increased to 10% body weight (6 weeks after the operation), 25% body weight, 50% body weight, and gradually reached 100% body weight (9-10 weeks after the surgery).

Clinical efficacy of the procedures were evaluated using a Hospital for Special Surgery (HSS) Knee scoring system^6^, the total score was 100, a score ≥ 90 indicated excellent efficacy, a score between 80 and 90 indicated good efficacy, a score between 70 and 80 indicated good efficacy, and a score < 70 indicated poor efficacy. We considered both excellent and good as excellent.

The degree of pain for all the patients was assessed six months after the operation based on a visual analogue scale (VAS); the higher the score, the more severe the pain. The Lysholm knee score was used to evaluate the function of the knee joint with higher score indicating better function of knee joint. Knee joint function recovery was evaluated by measuring the postoperative knee flexion angle (range of motion, ROM), with higher score indicating better knee joint function. SF-36 body score (SF-36p) was used to evaluate the recovery, with the higher score indicating better function of the knee joint. SF-36 psychological (SF-36M) score was used to evaluate the psychological recovery of patients after the operation, the higher the score, the better the knee function.

### Statistical Methods

Statistical analyses using the SPSS22.0 software was done. Measurement data were expressed as means and standard deviations (*x* ± s). Two samples between groups were compared using the *T* test and a single factor analysis of variance was performed to compare multiple groups. We expressed enumeration data as percentages and χ^2^ test results. A difference of *P*< 0.05 as statistically significant.

## RESULTS

As shown in Table-II, six months after operation, the percentage of patients with excellent and good scores in the suprapatellar approach group was 90.91% (50 out of 55), significantly higher than the percentage in the infrapatellar approach group, 76.36% (43 out of 55), P =0.038.

**Table II:** Comparison of clinical efficacy between the two groups [n (%)].

*Group*	*n*	*Excellent*	*Good*	*Medium*	*Poor*	*Excellent efficacy rate [n (%)]*
Infrapatellar approach group	55	24	18	7	6	42 (76.36)
Suprapatellar approach group	55	28	22	3	2	50 (90.91)
χ^2^value						4.251
P value						0.038

Clinical efficacy of the procedures was evaluated using an HSS scoring system. Score ≥ 90 indicates excellent efficacy, 80-90 indicates good efficacy, 70-80 indicates good efficacy, and a score < 70 indicates poor efficacy. Data are expressed as percentages and χ^2^ test results.

VAS score of the patients in the suprapatellar approach group six months after the procedure was 0.63±0.17, over 2-fold lower than that of the patients in the infrapatellar approach group 1.50±0.36 (P < 0.001). The Lysholm knee score was significantly higher in the suprapatellar group (78.92±6.25) as compared to the infrapatellar approach group (68.33±5.36), P<0.001. Similarly, ROM, SF-36p, and SF-36M scores were significantly higher than those in the infrapatellar approach group (P > 0.05) (Table-III).

**Table III:** Comparison of VAS, Lysholm knee, ROM, SF-36p, and SF-36M scores between the two groups at 6 months after operation (*x* ± sd).

*Group*	*n*	*VAS Scoring*	*Lysholm knee score*	*ROM*	*SF-36p score*	*SF-36M score*
Infrapatellar approach group	55	1.50±0.36	68.33±5.36	46.09±4.22	40.98±5.11	45.23±6.21
Suprapatellar approach group	55	0.63±0.17	78.92±6.25	55.34±4.02	48.19±6.42	49.22±5.06
χ2 value		16.206	9.539	11.770	6.517	3.694
P value		<0.001	<0.001	<0.001	<0.001	<0.001

Measurement data are expressed as means and standard deviations (x ± s). Enumeration data are expressed as χ^2^ test results.

## DISCUSSION

Closed reduction and intramedullary nailing is the main treatment for tibial shaft fractures. Commonly used tibial intramedullary nailing operations include infrapatellar and suprapatellar approaches. An inferior patellar tibial interlocking nail is commonly used. During the operation, the patient needs to have the knee joint bent at 90 degrees, which can easily lead to secondary fracture. In addition, the risk of fracture end displacement is increased.^7^,^8^ The surgical suprapatellar approach is new, it requires only 10 to 30 degrees of knee flexion and the probability of secondary injury is low. Moreover, intraoperative fluoroscopy is convenient and the direction of the needle is clear, making this approach close to being a minimally invasive procedure.^9^,^10^

The results of our study show that 6 months after the operation, the rate of patients with excellent efficacy in the suprapatellar approach group was significantly higher than that in the infrapatellar approach group. At the same time, the Vas score was significantly lower, and the Lysholm knee, ROM, SF-36p, and SF-36M scores were significantly higher in the suprapatellar approach than those in the infrapatellar approach group, suggesting superior curative effect of the suprapatellar procedure. Patients had overall less pain and faster recovery of the knee function after this type of operation. This may be due to the knee flexion to 90 degrees in the infrapatellar approach, stretching the quadriceps and patellar ligament of the affected limb, increasing the probability of ligament and muscle injury, and increasing the risks of fracture “secondary displacement” and of secondary nerve and vascular injuries (probably associated with anterior knee pain).^11^ The limb flexion angle during the suprapatellar approach is small, the fracture reduction and maintenance are easily achieved, and an intraoperative intramedullary nail can be inserted in a planned direction avoiding soft tissue injury. Moreover, the postoperative pain is little compared to that after the infrapatellar approach.^12^

The knee is an important weight-bearing joint, and knee function damage seriously affects the living standard of patients; thus, knee function recovery is a good indicator of the efficacies of the infrapatellar and suprapatellar approaches.^13^ The infrapatellar approach can cause a split ligament, weaken the quadriceps, create a callus at the intramedullary nailing site, cause pain by rubbing against the ligament, and cause secondary injury (all factors detrimental to the patient’s recovery).^14^ Intramedullary nailing through the infrapatellar approach can increase the pressure on the articular surface of the patient’s bone, cause abnormal stress on the articular cartilage, and lead to claudication.^15^ During the suprapatellar approach the damage to the infrapatellar soft tissue is minimal, and the integrity of the patient’s patellar ligament can be maintained, reducing postoperative knee pain and resulting in a fast postoperative recovery of the functional exercise capacity, promoting full knee function recovery.^9^ Our results show that the Lysholm knee, ROM, SF-36p, and SF-36M scores were significantly higher in the patients of the suprapatellar approach group than those in the patients of the infrapatellar approach group 6 months after operation, suggesting that the suprapatellar approach helps the recovery of the knee function.

### Limitations of the study

The main limitation of this retrospective study is that patients were only followed up for a duration of six months. As a result, longer-term effects for the suprapatellar and infrapatellar approaches could not be assessed from the data. Prospective and retrospective studies with larger sample size and longer follow up are needed to further evaluate the efficiency of both approaches.

## CONCLUSION

The clinical efficacy of the suprapatellar approach was significantly higher than that of the infrapatellar approach, and the suprapatellar approach can significantly reduce the degree of pain, promote the recovery of patients with knee joint involvement, improve the physical and psychological well-being, reduce the number of cases of postoperative delayed healing.

### Authors’ contributions

**ZZ:** Designed the project.

**ZW, PZ & XW:** Were involved in data collection and data analysis.

**ZZ:** Prepared the manuscript, approved the final version.

**JG:** Edited the manuscript.

**JG:** Is responsible and accountable for the accuracy or integrity of the work.

All authors read and approved the final manuscript.
